# Equine pituitary pars intermedia dysfunction: a spontaneous model of synucleinopathy

**DOI:** 10.1038/s41598-021-95396-7

**Published:** 2021-08-06

**Authors:** Jessica S. Fortin, Ashley A. Hetak, Kelsey E. Duggan, Caroline M. Burglass, Hailey B. Penticoff, Harold C. Schott

**Affiliations:** 1 Department of Pathobiology and Diagnostic Investigation, College of Veterinary Medicine, Michigan State University, East Lansing, MI 48824 USA; 2 Department of Large Animal Clinical Sciences, College of Veterinary Medicine, Michigan State University, East Lansing, MI 48824 USA

**Keywords:** Protein aggregation, Pituitary diseases, Parkinson's disease, Transmission electron microscopy

## Abstract

Equine pituitary pars intermedia dysfunction (PPID) is a common endocrine disease of aged horses that shows a similar pathophysiology as Parkinson’s Disease (PD) with increased levels of α-synuclein (α-syn). While α-syn is thought to play a pathogenic role in horses with PPID, it is unclear if α-syn is also misfolded in the pars intermedia and could similarly promote self-aggregation and propagation. Consequently, α-syn was isolated from the pars intermedia from groups of healthy young and aged horses, and aged PPID-afflicted horses. Seeding experiments confirmed the prion-like properties of α-syn isolated from PPID-afflicted horses. Next, detection of α-syn fibrils in pars intermedia via transmission electron microscopy (TEM) was exclusive to PPID-afflicted horses. A bank of fragment peptides was designed to further characterize equine α-syn misfolding. Region 62–87 of equine and human α-syn peptides was found to be most prone to aggregation according to Tango bioinformatic program and kinetics of aggregation via a thioflavin T fluorescence assay. In both species, fragment peptide 62–87 is capable of generating mature fibrils as demonstrated by TEM. The combined animal, bioinformatic, and biophysical studies provide evidence that equine α-syn is misfolded in PPID horses.

## Introduction

Parkinson’s disease (PD) affects more than 10 million people worldwide^1^. Despite considerable efforts to develop effective interventions, prevalence of this disease is expected to rise in the coming decades due to an aging population^1^. As a consequence, there is growing interest to develop, characterize, and validate novel, unconventional or innovative, non-rodent mammalian models of neurodegenerative diseases to replace transgenic rodent, fly, and worm models that have proven to be of poor predictive value in human clinical trials^2–15^. New models should replicate molecular, cellular, neuropathological, behavioral, and/or cognitive aspects of the neurodegenerative disease of interest^2–15^. Novel models could be essential tools to identify gaps in current knowledge of molecular mechanisms of neurodegenerative diseases, validate diagnostic modalities and develop new therapies.

Pituitary pars intermedia dysfunction (PPID)^16^, also known as Equine’s Cushing’s disease, is a common endocrine disease of aged horses that appears to have a pathophysiology similar to PD, with increased levels of α-synuclein (α-syn), possibly leading to a loss of dopaminergic neuron^17,18^. Increased amounts of α-syn have been found in histological sections of the pars intermedia of the pituitary gland of PPID-affected horses, as compared to young or aged non-PPID-affected horses^17^. In mammals, α-syn is a relatively well conserved and natively unfolded, monomeric protein found in presynaptic nerve terminals of dopaminergic neurons, with evidence of a role in modulating dopamine release^19,21^. Cellular environments that have high α-syn concentration, oxidative stress, phosphorylation, or nitration can promote α-syn protein misfolding, oligomerization, and aggregation that can disrupt cell functions and lead to neuronal cell death^22^. Accumulation of α-syn aggregates, coupled with chronic oxidative stress, can lead to a significant loss of dopaminergic neurons, a hallmark of neurodegenerative diseases including PD^23^. In sporadic PD, the characteristic motor dysfunction is due to the loss of dopaminergic inhibition in the substantia nigra due to accumulation of misfolded and aggregated α-syn^24^.

In PPID-affected horses, progressive loss of dopaminergic inhibition from the PVN leads to uncontrolled melanotrope proliferation in the pars intermedia of equine pituitary glands^16,25^. Progressive loss of dopaminergic inhibition is supported by increased plasma concentrations of pars intermedia-derived pro-opiomelanocortin (POMC) peptides, including adrenocorticotropin (ACTH), α-melanocyte stimulating hormone and β-endorphin^16,25–28^. Clinical signs of PPID include hypertrichosis, muscle wasting, abnormal fat distribution, bacterial infections, intestinal parasitism, lethargy, polyuria/polydipsia (PU/PD, laminitis, and variable neurological deficits^16,18,25,29–31^. At post-mortem histological examination, the pars intermedia of PPID-affected horses exhibits varying degrees of melanocyte hyperplasia and adenoma formation^28,32^.

Similarities between PD and PPID suggest that horses with PPID could be a suitable animal model for study of synucleinopathies. Horses have a longer lifespan than rodents. Because both diseases share aged-related risk factors and progression accelerates with age, using animals with shorter lifespans as models for neurodegenerative diseases has produced misleading conclusions^33^. In this study, we isolated α-syn from the pars intermedia of pituitary glands collected from young, aged non-PPID-affected, and aged PPID-affected horses to further understand the pathophysiology of equine PPID. We demonstrated that equine α-syn extracted from pituitary glands of horses with PPID is misfolded. Tango bioinformatic program analyses indicated that equine α-syn had a propensity to aggregate and we found that residues 62–87 of both equine and human α-syn had the highest propensity to aggregate. This peptide fragment is part of the non-amyloid-β component (NAC) region of α-syn, associated with oligomer formation, which may lead to the cytotoxicity of dopaminergic neurons in PD and PPID. Our data demonstrate that equine α-syn is misfolded in PPID-affected horses. Thus, horses with PPID could represent a novel model for study of synucleinopathies that might allow identification of new protein biomarkers for diagnostic purposes or investigation of novel therapeutics for PD.

## Results

### PPID case confirmation

This study investigated misfolding of α-syn in the pars intermedia collected from horses of different ages and disease status to provide further insight into the pathophysiology of equine PPID. Three groups of horses (n = 5 in each group) were studied: young horses, healthy aged horses without PPID, and aged horses with PPID. All horses with PPID had supportive clinical signs (primarily hypertrichosis) and enlarged PGs with a histologic PG score of 5/5 (Table 1).

**Table 1:** Mean age and pituitary gland weights and histologic grades for each group of horses.

Groups of horses (n)	Age mean (year) ± SD	Pituitary gland weight mean (g) ± SD	Histologic grade median ± range
Young (5)	6 ± 3	2.1 ± 0.4	2 ± 1
Aged (5)	29 ± 5	1.9 ± 0.3	4 ± 1
PPID (5)	27 ± 4	5.7 ± 1.2	5 ± 0

### α-Syn extracted from pars intermedia tissue of PPID-affected horses promotes human α-syn fibril formation

**Figure 1:**
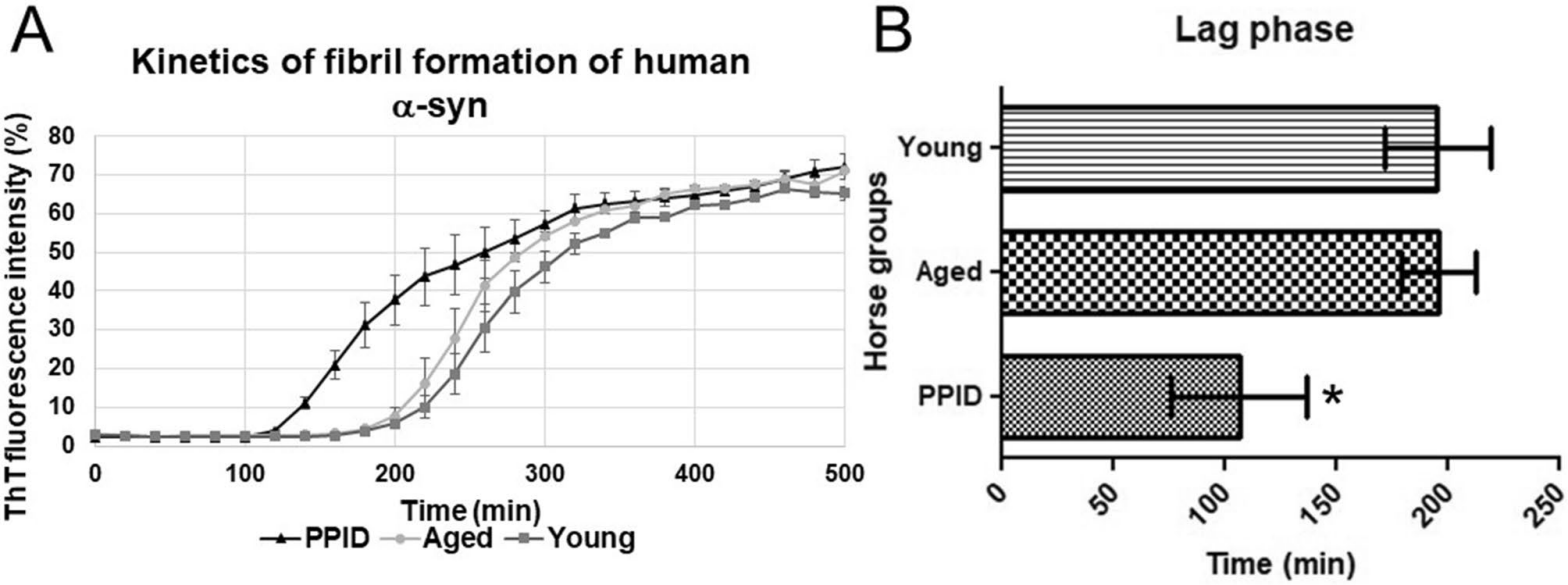
Alpha-synuclein (α-syn) extracted from the pars intermedia of PPID-affected horses has the potential to cross seed recombinant human α-syn. (**A**) Thioflavin T (ThT) fluorescence assay kinetics of human α-syn fibril formation in the presence of equine α-syn isolated from young, aged, and PPID-affected horses. Data represent the mean of the fluorescence intensity with error bars for SEM obtained from three different horses in each group (young, aged, PPID). (**B**) Lag phase time (min) for initiation of human α-syn fibril formation during incubation with equine α-syn extracted from pars intermedia tissue collected from young, aged and PPID-affected horses. The time (min) required for human α-syn fibril elongation (lag phase) is shorter when induced with equine α-syn extracted from PPID-affected horses. Data represent the mean of the lag phase time (min) obtained from five different horses in each group (young, aged, PPID). Data were analyzed by the one-way analysis of variance with Dunnett's multiple comparison post-hoc testing between groups. (*significant difference p < 0.05.)

Our work focused on detection of α-syn aggregates in the pars intermedia collected from young, aged and PPID-affected horses, where dopaminergic axons project into this important part of the pituitary gland. We first examined the propensity of equine α-syn isolated from the pars intermedia of controls (healthy young and aged horses) and PPID-affected horses to induce the aggregation of human α-syn recombinant protein. Seeding assays performed with immunoprecipitated equine α-syn co-incubated with human recombinant α-syn, using thioflavin T (ThT) as a probe to follow kinetics of fibril formation^34^, demonstrated that equine α-syn extracted from PPID-affected horses significantly reduced the time required for human α-syn fibril formation (lag time), as compared to pars intermedia extracts collected from young and aged horses (Fig. 1). This finding strongly suggests presence of misfolded equine α-syn in PPID-affected horses that is capable to act as a template and cross-seed human α-syn fibrillization.

### α-Syn extracted from pars intermedia tissue of PPID-affected horses exhibits a fibrillary ultrastructure

**Figure 2:**
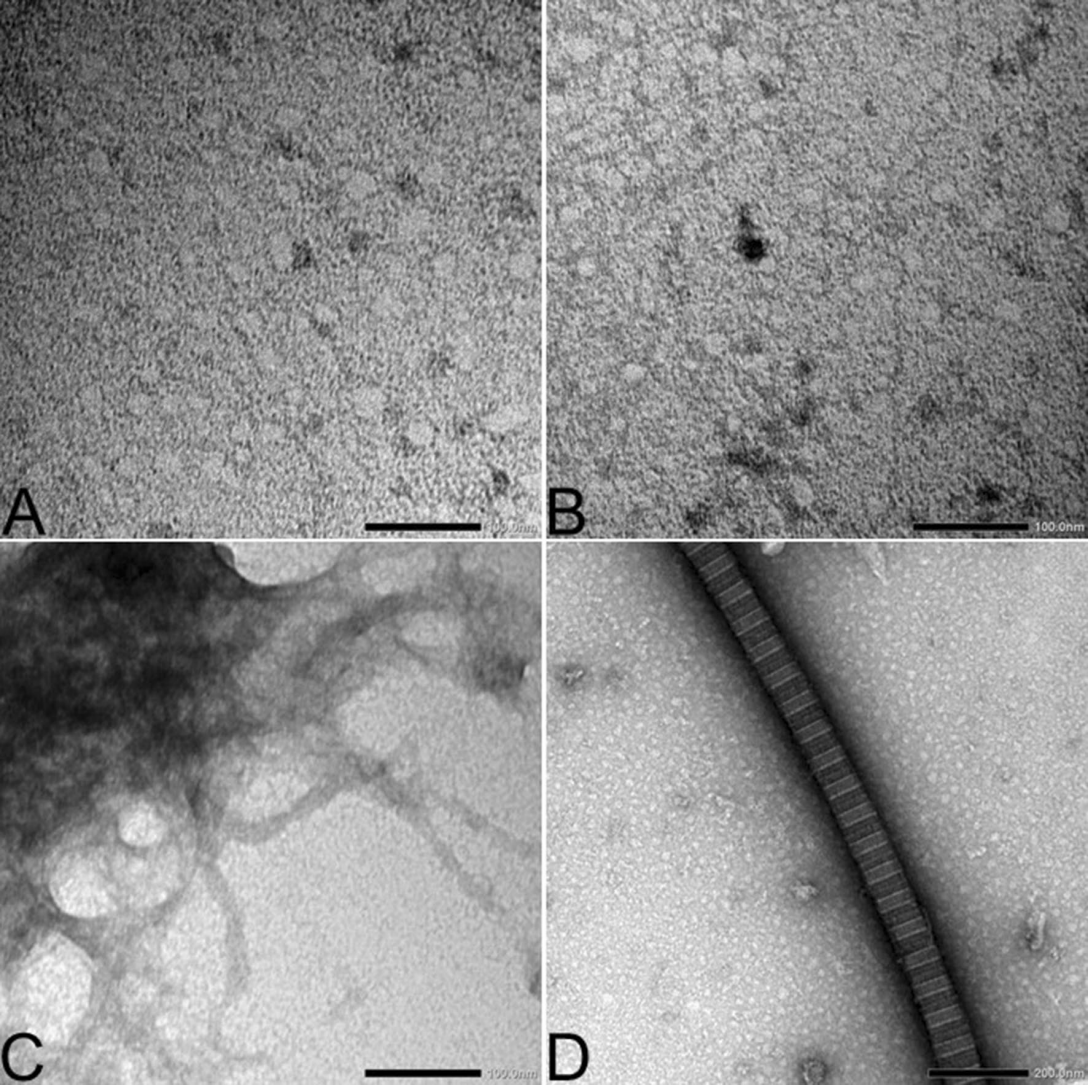
Alpha-synuclein (α-syn) extracted from the pars intermedia of PPID-affected horses exhibits a fibrillar ultrastructure as visualized by transmission electron microscopy (TEM). (**A**) TEM image of the background staining showing the absence of fibrils in equine α-syn isolated by immunoprecipitation (IP) from the pars intermedia of a young horse. (Scale bar, 100 nm.) (**B**) TEM image of α-syn isolated by IP from pars intermedia of an aged horse representing background staining (no fibrils). (Scale bar, 100 nm.) (**C**) TEM image of positively stained equine α-syn fibrils isolated by IP from the pars intermedia of a PPID-affected horse. (Scale bar, 100 nm.) (**D**) TEM image of a collagen fiber. (Scale bar, 200 nm.)

To validate seeding assay results, we compared the ultrastructure of equine α-syn isolated from the pituitary gland of young, aged and PPID horses by transmission electron microscopy. No fibrils were detected in immunoprecipitated α-syn obtained from the pars intermedia of young and aged horses (Fig. 2A,B). α-syn extracted from the pituitary gland of all PPID-affected horses contained a few mature fibrils on the copper grid. Fibrils were 7 to 10 nm in diameter and were characteristic of amyloid fibrils (Fig. 2C). Rare large fibrils (> 100 nm in diameter) with cross-striation were found in all pars intermedia samples, consistent with collagen (Fig. 2D).

**Figure 3:**
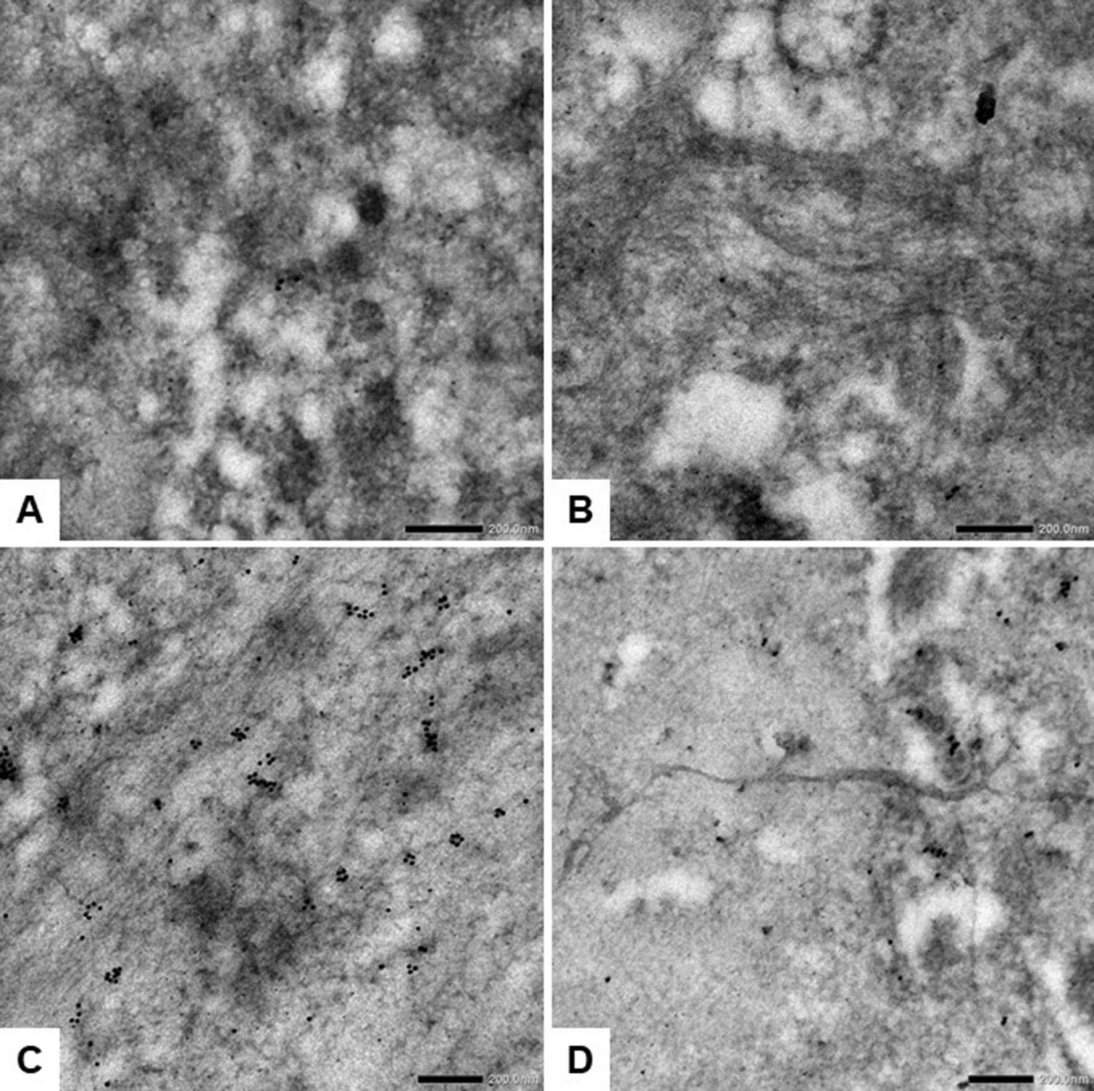
Immunogold transmission electron microscopy (TEM) using an α-synuclein primary antibody on pars intermedia tissue confirms presence of fibrils in a PPID-affected horse. (**A**) TEM image from the pars intermedia of an PPID-affected horse without use of the primary antibody consists of background staining of the secondary antibody. (**B**) TEM image of the pars intermedia of an aged horse shows an immunogold labeling similar to background straining. (**C**) TEM image of the pars intermedia of a PPID-affected horse exhibits immunogold labeling. (**D**) TEM image of the pars intermedia of a PPID-affected horse contains a fibril with minimal immunogold labeling in the extracellular milieu (Scale bars, 200 nm).

To confirm the presence of fibrils in the pituitary gland of PPID-affected horses, we then examined the ultrastructure of α-syn in the pars intermedia tissue of an aged horse and a PPID-affected horse using the immunogold technique (Fig. 3). Increased levels of immunogold were detected in the pars intermedia of the PPID-affected horse (Fig. 3C) in comparison with the aged horse (Fig. 3B). One fibril was detected extracellularly in the pars intermedia section collected from the PPID-affected horse (Fig. 3D). This fibril was immunogold positive for α-syn in few areas. Background staining of the pars intermedia in the absence of primary antibody was low (Fig. 3A).

### Equine α-syn synthetic fragment peptide 62–86 is highly prone to aggregate

**Figure 4:**
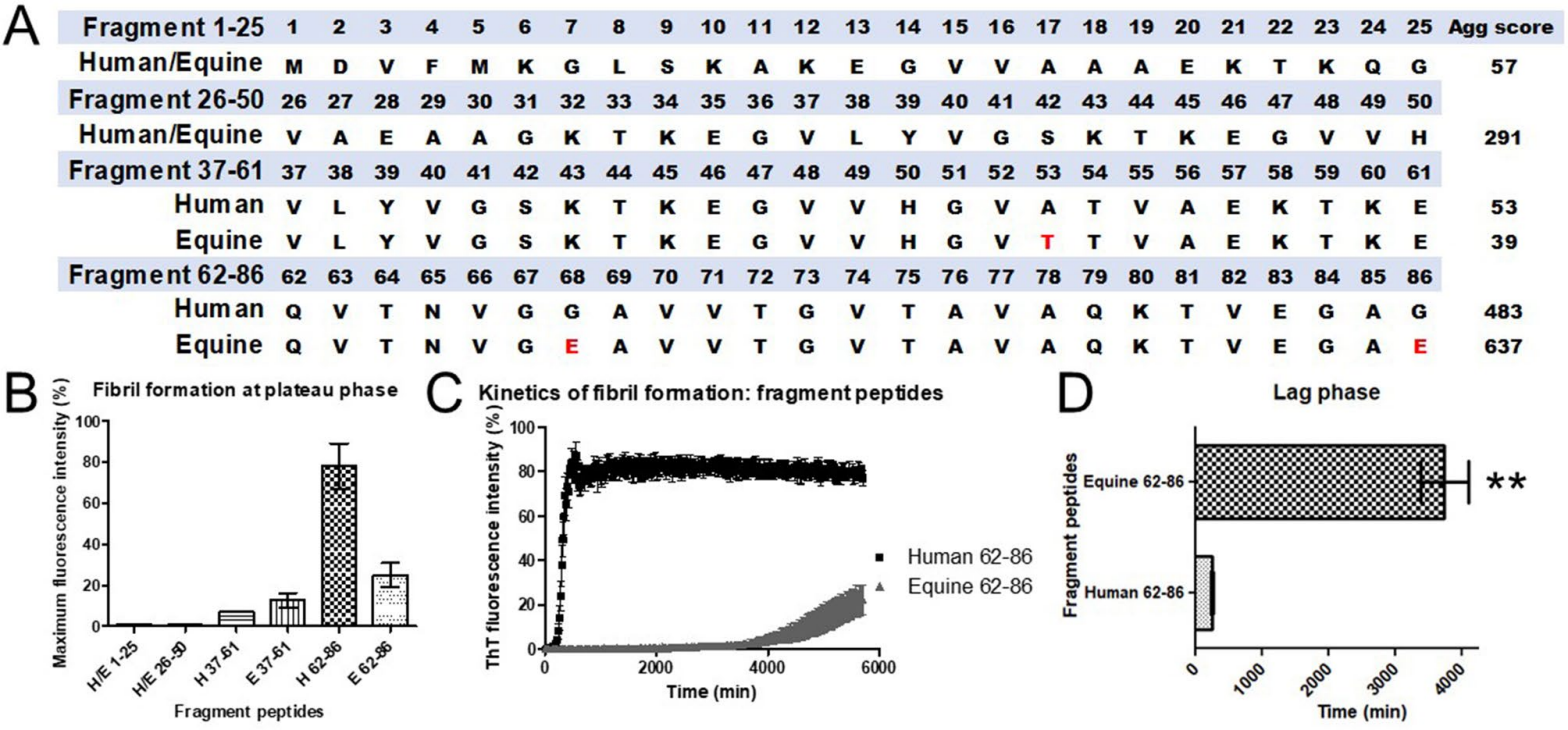
Equine α-synuclein (α-syn) fragment peptide 62–86 generated fibrils after a longer lag phase period in comparison with its human counterpart using ThT binding assays. (**A**) Sequence of α-syn synthetic fragment peptides used to investigate aggregation propensity of equine α-syn. Aggregation scores determined by Tango bioinformatic program are provided in the right-sided column for each peptide fragment. Fragments exhibiting an aggregation score of 30–60 were selected as negative controls. Fragments with scores near or above 300 were further analyzed with biophysical assays to study the propensity of aggregation in vitro. (**B**) The maximum of fluorescence intensity at plateau phase of selected fragment peptides: bars show mean of fluorescence with error bars for SEM of three replicates. (**C**) Kinetics of α-syn fibril formation of human and equine fragment peptides 62–86. Data represent the mean of the fluorescence intensity with error bars for SEM obtained from three replicates. (**D**) Lag time required for fibril elongation of equine fragment peptide 62–86 is longer than for the human fragment peptide 62–86. Two-way analysis of variance with Bonferonni post-hoc testing to compare replicate means by row. Differences were considered statistically significant at * p < 0.05 and ** p < 0.01.

We further examined kinetics of fibril formation of human and equine α-syn synthetic fragment peptides to validate the propensity of equine α-syn to aggregate. We first compared full length α-syn amino acid sequences (140 amino acids) of both species, which indicated seven amino acid variations in the peptide sequence (A53T, G68E, G86E, V95G, Q99H, N103S, P108A). We used the bioinformatic Tango program (35–37) to predict an aggregation score and regions rich in β-sheets to design fragment peptides. The aggregation score (Agg score) resulting from full length α-syn sequences indicated that equine α-syn was highly prone to aggregate with an Agg score of 1042, as compared to an Agg score of 896 for human α-syn. We subsequently truncated α-syn (full length of 140 amino acids) into 25 amino acid fragments to identify regions most susceptible to aggregation (Table 2). To design the synthetic fragment peptides, specific regions were selected as representative fragments with null (regions 1–25 and 26–50), low (region 37–61), and high (region 62–86) propensity to aggregate (Fig. 4A).

**Table 2:** Human (*Homo sapiens*) and horse (*Equus caballus*) α-synuclein (α-syn) amino acid sequences. The 140 amino acid α-syn protein consists of three regions: N-terminal amphipathic region (residues 1 to 60), hydrophobic non-amyloid-β component (NAC) (residues 61–95) and the acidic C-terminal region (residues 96–140). Tango bioinformatic aggregation scores (35–37) of the full-length human and equine α-syn are 896 and 1042, respectively. Aggregation scores for each segment of the human and equine α-syn.

Fragment	1	2	3	4	5	6	7	8	9	10	11	12	13	14	15	16	17	18	19	20	21	22	23	24	25	Variation	Agg score
Human	M	D	V	F	M	K	G	L	S	K	A	K	E	G	V	V	A	A	A	E	K	T	K	Q	G		57
Horse	M	D	V	F	M	K	G	L	S	K	A	K	E	G	V	V	A	A	A	E	K	T	K	Q	G	0	57
Fragment	26	27	28	29	30	31	32	33	34	35	36	37	38	39	40	41	42	43	44	45	46	47	48	49	50		
Human	V	A	E	A	A	G	K	T	K	E	G	V	L	Y	V	G	S	K	T	K	E	G	V	V	H		291
Horse	V	A	E	A	A	G	K	T	K	E	G	V	L	Y	V	G	S	K	T	K	E	G	V	V	H	0	291
Fragment	51	52	53	54	55	56	57	58	59	60	61	62	63	64	65	66	67	68	69	70	71	72	73	74	75		
Human	G	V	A	T	V	A	E	K	T	K	E	Q	V	T	N	V	G	G	A	V	V	T	G	V	T		117
Horse	G	V	T	T	V	A	E	K	T	K	E	Q	V	T	N	V	G	E	A	V	V	T	G	V	T	2	56
Fragment	76	77	78	79	80	81	82	83	84	85	86	87	88	89	90	91	92	93	94	95	96	97	98	99	100		
Human	A	V	A	Q	K	T	V	E	G	A	G	S	I	A	A	A	T	G	F	V	K	K	D	Q	L		5
Horse	A	V	A	Q	K	T	V	E	G	A	E	S	I	A	A	A	T	G	F	G	K	K	D	H	L	3	8
Fragment	37	38	39	40	41	42	43	44	45	46	47	48	49	50	51	52	53	54	55	56	57	58	59	60	61		
Human	V	L	Y	V	G	S	K	T	K	E	G	V	V	H	G	V	A	T	V	A	E	K	T	K	E		53
Horse	V	L	Y	V	G	S	K	T	K	E	G	V	V	H	G	V	T	T	V	A	E	K	T	K	E	1	39
Fragment	62	63	64	65	66	67	68	69	70	71	72	73	74	75	76	77	78	79	80	81	82	83	84	85	86		
Human	Q	V	T	N	V	G	G	A	V	V	T	G	V	T	A	V	A	Q	K	T	V	E	G	A	G		483
Horse	Q	V	T	N	V	G	E	A	V	V	T	G	V	T	A	V	A	Q	K	T	V	E	G	A	E	2	637
Fragment	91	92	93	94	95	96	97	98	99	100	101	102	103	104	105	106	107	108	109	110	111	112	113	114	115		
Human	A	T	G	F	V	K	K	D	Q	L	G	K	N	E	E	G	A	P	Q	E	G	I	L	E	D		0
Horse	A	T	G	F	G	K	K	D	H	L	G	K	S	E	E	G	A	A	Q	E	G	I	L	E	D	4	0
Fragment	116	117	118	119	120	121	122	123	124	125	126	127	128	129	130	131	132	133	134	135	136	137	138	139	140		
Human	M	P	V	D	P	D	N	E	A	Y	E	M	P	S	E	E	G	Y	Q	D	Y	E	P	E	A		0
Horse	M	P	V	D	P	D	N	E	A	Y	E	M	P	S	E	E	G	Y	Q	D	Y	E	P	E	A	0	0

Peptide are provided in the right-sided column.

Kinetics of fibril formation with each fragment peptide were assessed with ThT fluorescence assays and maximum fluorescence intensity obtained at the end of the kinetic assays was compared for each fragment (Fig. 4A-C). Region 1–25 and 26–50, identical in both species, exhibited a null Agg score (Fig. 4A) and did not generate fibrils during the ThT fluorescence assays (Fig. 4B). However, fluorescence signals were increased with the truncated equine and human α-syn peptides representing regions 37–61 and 62–86, with region 62–86 being highly susceptible to aggregate. Aggregation of equine α-syn fragment 62–86 required more time for elongation as compared to its human counterpart (Fig. 4C). The highest fluorescence intensity was found with α-syn fragment 62–86 in both species, corroborating the highest Agg score determined by Tango bioinformatic program.

**Figure 5:**
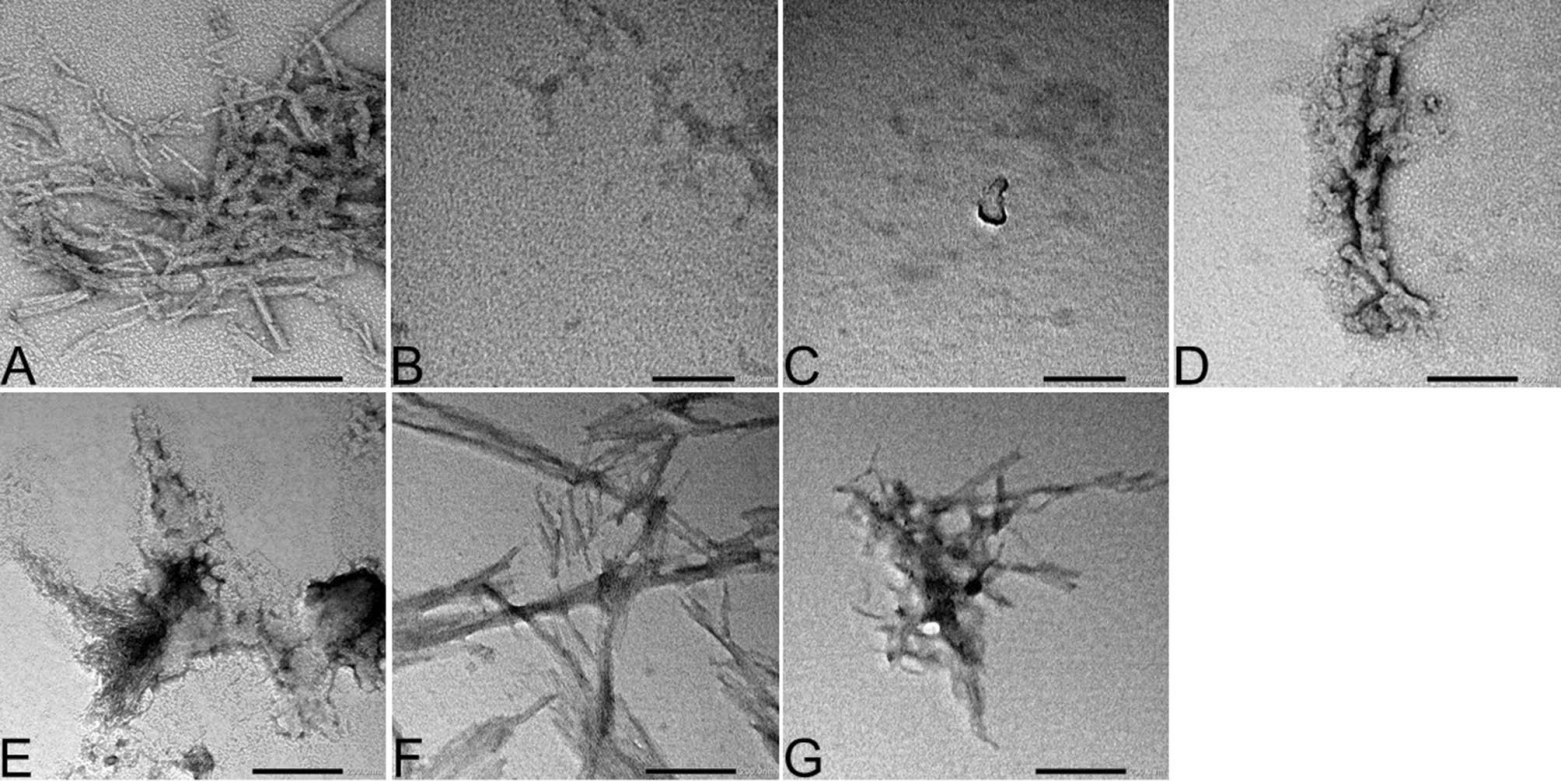
Transmission electron microscopic (TEM) images of human and equine α-synuclein (α-syn) peptide fragments at the end of kinetics of fibril formation (after 96 h of incubation at 37 °C). (**A**) TEM images of human α-syn full-length recombinant peptide at the end of the kinetics of aggregation. (Scale bar, 200 nm.) (**B**) TEM image of human and equine α-syn synthetic fragment 1–25. (Scale bar, 100 nm.) (**C**) TEM image of human and equine α-syn synthetic fragment 26–50. (Scale bar, 100 nm.) (**D**) TEM image of human α-syn synthetic fragment 37–61. (Scale bar, 200 nm.) (**E**) TEM image of equine α-syn synthetic fragment 37–61. (Scale bar, 200 nm.) (**F**) TEM image of human α-syn synthetic fragment 62–86. (Scale bar, 200 nm.) (**G**) TEM image of equine α-syn synthetic fragment 62–86. (Scale bar, 200 nm.)

Electron microscopy is the gold-standard method used to detect presence of fibrils at the end of the kinetic studies^34^. All samples were visualized on grids via transmission electron microscopy and confirmed fibril formation with fragments 37–61 and 62–86 and lack of fibrils with fragments 1–25 and 1–50 (Fig. 5). Equine α-syn peptide fragments were capable of forming fibrils under the same conditions as human α-syn fragments and full length α-syn. Interestingly, numerous globular structures surrounded short fibrils of equine and human α-syn fragment 37–61, suggestive of oligomerization (Fig. 5D-E).

## Discussion

Horses with PPID have been shown to have higher levels of α-syn in dopaminergic nerve terminals within the pars intermedia compared to healthy aged horses^17^. In addition to overexpression in PPID-affected horses, α-syn co-localized with increased 3-nitrotyrosine immunostaining in pars intermedia tissue, supporting a pathophysiologic role of oxidative damage^17^. Equine PPID exhibits neurochemical perturbations including oxidative stress and antioxidant deficiency that may provide a favorable environment for protein aggregation^38^. Previous work demonstrated reduced dopamine content in pars intermedia of PPID-afflicted horses^17,33,38,39^. The cell bodies of the dopaminergic neurons that innervate the pars intermedia originate from the periventricular nuclei^39–41^. In PD, damaged dopaminergic neurons arise in the substantia nigra compacta which degenerate and cause dopamine loss in the basal ganglia, crucial for coordination of fine motor movements^42^. Although the affected dopaminergic neurons in PPID horse are located at a different site than in PD, misfolding of α-syn may play a similar pathophysiological role. Our study provides evidence that α-syn is misfolded (i.e. accumulation of fibrils) in PPID horses. Additional studies will be required to determine if misfolded α-syn is a contributor or a consequence of the neurodegeneration of dopaminergic neurons in PPID horses. The neurodegeneration of dopaminergic neuron in PPID horses is assumed due to reduction of both tyrosine hydroxylase and its product dopamine in the pars intermedia of affected horses^17^. The clinical response of affected horses to dopamine agonist therapy provides indirect evidence of neurodegeneration^25^. However, additional studies would be important to confirm if misfolded α-syn can lead to loss of dopaminergic neurons in PPID-affected horses.

In a prion-like fashion, α-syn is capable of self-propagation within neurons and seeds monomeric α-syn^43^. This mechanism is thought to play a pivotal role in neurodegeneration of dopaminergic neurons in PD. To investigate whether α-syn isolated from PPID-affected horses has the propensity to seed human α-syn, we co-incubated the immunoprecipitated α-syn from pars intermedia of young, aged and PPID-affected horses with recombinant human α-syn. The immunoprecipitated α-syn from pars intermedia of PPID-affected horses shortened the time required for initiation of in vitro fibril elongation of recombinant human α-syn in comparison to α-syn isolated from young and aged horses. A shorter lag time with immunoprecipitated equine α-syn from the PPID group indicates that equine α-syn collected from PPID-affected horses exhibits an aberrant and/or misfolded conformation that is capable of cross-seeding human α-syn. In the immunoprecipitated samples, we detected fibrils exclusively in the pars intermedia tissue collected from PPID-affected horses. We confirmed this result by performing an immunogold TEM experiment using tissue sections from the pars intermedia of an aged healthy horse and a PPID-affected horse. Interestingly, human α-syn fibrils have been shown to be 1,000-fold more toxic to cells^44^. Whether α-syn oligomers are more toxic than fibrils remain controversial. For this reason, the present study did not focus on tracking the oligomers in the pars intermedia tissue.

To validate the propensity of equine α-syn to misfold, we analyzed regions of the peptide sequence. The equine α-syn peptide sequence has seven residue variations in comparison to its human counterpart. Interestingly, equine α-syn amino acid sequence exhibits the A53T variation, which represents one of the five point mutations (A30P, E46K, H50Q, G51D and A53T) associated with early onset PD^45–51^. One hypothesis to explain the pathogenicity of α-syn harboring mutations A53T and/or E46K is a decrease in tetrameric α-syn conformation and an increase in α-syn monomeric conformation which generates unfolded α-syn monomer as a potential source of toxicity^52^. Paradoxically, equine α-syn contains three amino acid variations (i.e. A53T, G86E, V95G) that have been shown to weaken fibrillization and possible toxicity using anionic lipid vesicles^53^. The clinical relevance of this finding needs to be clarified further. The kinetics of aggregation in our study showed that the equine fragment peptide 62–87 took more time to aggregate, in comparison with the human α-syn fragment, which might corroborate with the anionic lipid vesicle kinetic studies performed with recombinant elephant, pig, and whale α-syn^53^.

The in silico analysis of human and equine α-syn allowed us to design truncated peptides of 25 amino acids and identify areas most highly susceptible to aggregation. The most critical region identified contained amino acids 62 to 86. We opted for design of synthetic fragment peptides as a strategy to correlate the aggregation propensity of equine α-syn observed during the seeding experiments and visualization of fibrils by electron microscopy of the immunoprecipitated equine α-syn sample from pars intermedia tissue collected from a PPID-affected horse. In addition, fragment peptides and mutant peptides can pinpoint critical residues involved for oligomer and fibril formation^36,37,54^. The kinetics of aggregation resulting from the selected set of fragments confirmed that region 62–86 in both human and equine α-syn is highly prone to aggregate into mature fibrils. Fragment 62–86 is part of the NAC, a hydrophobic region that may contribute to α-syn oligomerization^43^. Interestingly, globular structures with fewer fibrils were observed by electron microscopy of human and equine fragments 37–61. Further studies will be needed to confirm the importance of amino acids at the proximal region of the NAC, where residue 61 may have an important role in oligomerization. The carboxy-terminal region (90–140) exhibited the lowest aggregation score and was not investigated further in this study. This region is intrinsically disordered and likely contributes minimally to aggregation^55^. We selected instead regions 1–25 and 26–50 as negative controls for aggregation.

Taken together, we demonstrated that α-syn is misfolded in the pars intermedia tissue of PPID-affected horses by documenting presence of fibrils in both the pars intermedia protein extract and tissue section. α-Syn isolated from these PPID-affected horses has the ability to reduce the lag time of human α-syn fibrillization in contrast to α-syn isolated from healthy young and aged horses. In addition, our findings indicate the importance of some amino acids responsible for equine α-syn aggregation located at the end of the N-terminal amphipathic proximal region and involving the proximal NAC region. Understanding the conformational state of α-syn in equine PPID provides an opportunity for establishing a unique non-rodent mammalian model of PD. Naturally occurring neurodegenerative mammalian models are extremely relevant to validate new diagnostic methods and to identify new therapeutic targets that could modify disease progression by limiting conversion of α-syn into its toxic form, and preventing further dopaminergic neurodegeneration characteristic of both PPID and PD. The present study provides a solid foundation to pursue equine PPID as an animal model of neurodegeneration to identify early diagnostic tests and to provide new therapeutic approach(es) preventing dopaminergic neurodegeneration.

## Materials and Methods

### Animals and tissue collection

Study groups consisted of fifteen horses presented to MSU VMC for elective euthanasia. Groups were comprised of five healthy aged horses, five aged horses with clinical signs (primarily hypertrichosis) of pituitary pars intermedia dysfunction (PPID) and five young horses. After clinical examination study horses were humanly euthanized with Na pentobarbital (100 mg/kg, IV). Pituitary glands were collected and sectioned along a midline sagittal plane within less than 1 h after euthanasia, as described^56^. One half of the pituitary gland was immediately placed on ice and frozen (−86 °C) and the other half was immersed in 100 mL of 10% neutral buffered formalin and fixed at room temperature for ≥ 72 h. All procedures were approved by the Michigan State University Institutional Animal Care and Use Committee (PROTO201900231). The study was carried out in compliance with ARRIVE guidelines. All methods were performed in accordance with relevant guidelines and regulations.

### Histologic examination

Sections of the pituitary gland were trimmed in longitudinal orientation, processed and embedded in paraffin blocks. 4 μm thick sections were cut from each block using a microtome and captured on charged slides. H&E stained sections of the pituitary gland were examined to document PPID histological score and to localize the pars intermedia prior to dissection of the contralateral frozen specimens^32^. The histological grading system used to evaluate the pituitary glands was the following: grade 1, normal; grade 2, focal or multifocal pars intermedia hypertrophy or hyperplasia; grade 3, diffuse pars intermedia adenomatous hyperplasia; grade 4, pars intermedia adenomatous hyperplasia with microadenomas (1–5 mm in diameter); grade 5, adenoma of more than 5 mm in diameter located in the pars intermedia or pars anterior^32^. Pituitary gland histological score was 5 in all PPID horses, as compared to median scores of 2 (range 2–4) and 2 (range 1–2) for aged and young horses, respectively (Table 1)^32^.

### Extraction of proteins

After histological localization, three samples (~ 35 mg each) of pars intermedia was dissected from each freshly thawed contralateral pituitary gland, assuming mirror-image localization of pars intermedia tissue. Specimens were lysed for 12 h at 4 °C in 500 μL RIPA sample buffer. To complete cell lysis, samples were sonicated and protein concentration was determined with a modified Lowry protein assay kit (Thermo Scientific, ref 23,240, Rockford, IL).

### Chemical and α-syn peptides

Thioflavin-T (ThT) was obtained from Alfa Aesar (Ward Hill, MA). Synthetic α-syn (140 amino acids) was obtained from rPeptide (Watkinsville, GA). Synthetic α-syn fragment peptides of 25 amino acids were purchased from GenScript (Piscataway, New Jersey).

### α-syn immunoprecipitation experiments

Pars intermedia protein extracts (250 µg) were dissolved in 500 µL of 10 mM Tris HCl (pH 8) supplemented with 1 mM MgCl_2_. Rabbit polyclonal anti-α-syn (4 µg) (Invitrogen, Thermo Fisher Scientific, PA5-85,343, Rockford, IL) was added and samples were incubated with mixing for 2 h at 4 °C. Protein A/G Plus-Agarose beads (40 μL) (sc-2003, Santa Cruz Biotechnology, Dallas, TX) were added and samples were incubated for 12 h at 4 °C with constant mixing. Sepharose beads were pelleted by centrifugation at 4 °C (10 min, 2,500 rpm) and washed three times with 0.5 mL of 10 mM Tris HCl (pH 8). Following the final wash, pellets were resuspended in 60 µL of 20 mM Tris–HCl (pH 7.4) supplemented with 100 mM NaCl. Controls consisted of 4 µg of rabbit IgG (BioVision, ref 1268–100, Milpitas, CA) or rabbit anti-α-syn (4 µg) with Protein A/G Plus-Agarose beads in the absence of protein extracts. After validation by western blot with anti-α-syn, immunoprecipitation was repeated twice and pellets were used for seeding experiments and transmission electron microscopy.

### Seeding experiments

A ThT dye fluorescence assay was performed using immunoprecipitated α-syn from all three groups of horses to evaluate kinetics of in vitro α-syn fibril formation. Synthetic human α-syn (rPeptide, Watkinsville, Georgia) was dissolved in 20 mM Tris–HCl (pH 7.4) supplemented with 100 mM NaCl) to a stock solution of 276 μM (1 mg in 250 μL). 7.25 μL of peptide was transferred to a black 384 well microplate with a flat bottom and medium binding (Greiner Bio-One, ref 784,076). Each well was filled with 13.25 μL of recombinant human α-syn at a concentration of 138 μM. Experiments were performed using a solution of ThT at a final concentration of 36 µM (3 μL in each well, stock 160 μM) with 3 μL immunoprecipitated α-syn resuspended in PBS from each group (preformed fibril from PPID, normal peptide from aged and young groups). Background signal consisted of assay buffer with ThT. The plate was sealed and fluorescence was measured in a Synergy HT multi-mode microplate reader (BioTek, Winooski, VT) in slow shaking mode for 30 s prior reading at 20 min intervals for 95 h at 37 °C with excitation and emission wavelengths set at 440 and 485 nm, respectively. Lag times were calculated with GraphPad Prism version 5.03 using the nonlinear fit data (plateau followed by one phase decay).

### Thioflavin T fluorescence experiment with fragment peptides

To validate aggregation propensity of equine α-syn, ThT-based fluorescence assays were used to detect formation of fibrils of human and equine α-syn fragment peptides. Each fragment peptide was dissolved in 20 mM Tris–HCl (pH 7.4) supplemented with 100 mM NaCl to a stock solution of 1 mM. ThT assays were performed in 10 mM PBS buffer (pH 7.4), supplemented with 0.5 mM SDS and 300 mM NaCl and transferred to a non-treated black 96 well microplate with a transparent flat bottom (Corning, ref 3631). Each well was filled with 150 μL buffer with a peptide concentration of 100 μM and one 3 mm borosilicate bead (Kimble®, Kimax, ref 13,500–3). Experiments with α-syn fragment peptides were initiated by adding ThT to achieve a final concentration of 20 µM ThT. The background signal consisted of ThT in buffer without α-syn fragment peptides. Fluorescence emission experiments were performed as described above for the seeding experiments. Measurements were taken at 37 °C every 20 min with 10 s prior shaking over 96 h. Three replicates of each fragment peptides were assayed and experiments were repeated three times using two independent α-syn fragment peptide stock solutions. For each time point, arbitrary fluorescence units were calculated from the mean values normalized to the maximum value in each completed assay. Arbitrarily, the maximum value for fluorescence intensity was established with human α-syn fragment 62–86. Lag times were calculated as previously described in seeding experiments.

### Transmission electron microscopy (TEM)

After α-syn was immunoprecipitated from pars intermedia tissue (all three groups) and after kinetics of fibril formation with synthetic fragment peptides was completed, 10 µL of fragment peptide was applied to a 400-mesh Formvar-carbon-coated copper grid (Electron Microscopy Sciences, Hatfield, PA) to confirm presence or absence of fibrils. Grids were incubated for 1 min and washed three times with distilled water. After air-drying, grids were incubated for 1 min in a fresh solution of 1% uranyl acetate. Grids were air-dried again and imaged via transmission electron microscopy (JEOL 1400 Flash, Japan). Pictures were acquired at an accelerating voltage of 100 kV and magnification of 40 and 80 k.

### Immunogold TEM

A small area (~ 0.5 mm^3^) of the pars intermedia was dissected and immersed in 5 ml of 10% neutral buffered formalin with 0.5% glutheraldehyde for 48 h at room temperature. The tissues were embedded in LR white (an acrylic resin). Sections were 70 nm in thickness and deposited on a formvar coated gold grid. For the immunogold, the grids were pre-incubated for 10 to 30 min with a blocking solution consisting of 0.01 M PBS, 5% BSA, and 5% normal goat serum. The grids were washed three times (5 min each) with the incubation buffer system (0.01 M PBS with 0.1% BSA). The grids were incubated with the primary antibody (rabbit polyclonal anti-α-syn, Invitrogen, Thermo Fisher Scientific, Rockford, IL) diluted at 1/10 in the incubation buffer system for 1 h at room temperature. After, the grids were transferred into the incubation buffer system for 5 min. This step was repeated two additional times. The grids were incubated with the secondary antibody (anti-rabbit, electron microscopy sciences) conjugated with 10 nm colloidal gold (diluted at 1/20 with the incubation buffer system) for 1 h at room temperature. The grids were washed three times (5 min each) with the incubation buffer system and three more times (5 min each) with doubled distilled water. Uranyl acetate at a concentration of 4% (for 2 min) and Reynolds lead citrate were used to stain the grids.

## Data Availability

All study data are included in the paper. Correspondence should be addressed to J.S.F. and H.C.S.
